# 2500. An Audit of Hepatocellular Carcinoma Surveillance amongst Patients with Chronic Hepatitis B Attending an Irish Tertiary Centre

**DOI:** 10.1093/ofid/ofad500.2118

**Published:** 2023-11-27

**Authors:** Aoife Heeney, Aine Ni Chianain, Cora McNally

**Affiliations:** Mater Misericordiae University Hospital, Dublin, Ireland, Dublin, Dublin, Ireland; Beaumont Hospital, Dublin, Ireland, Dublin, Dublin, Ireland; Beaumont Hospital, Dublin, Ireland, Dublin, Dublin, Ireland

## Abstract

**Background:**

The association between chronic hepatitis B (CHB) and hepatocellular carcinoma (HCC) is well established, however it can be challenging to identify those who require surveillance. The European Association for the Study of the Liver (EASL) guidelines recommend 6-monthly liver ultrasound (US) as the optimal surveillance method and using the PAGE-B score for HCC risk stratification. This score offers good predictability for HCC whilst on nucleoside analogue therapy, and it also appears reliable in untreated patients. It has also been validated for individuals with HIV/HBV coinfection. We aimed to assess the compliance of an Irish tertiary hospital with the EASL guidelines for HCC surveillance.

**Methods:**

We carried out a retrospective review of electronic medical records of patients with CHB attending the infectious diseases outpatient clinic in Beaumont Hospital, Dublin, Ireland.

**Results:**

70 patients were included, of which 20 were co-infected with HIV. Of the 50 patients with CHB monoinfection, 4% (n=2) had cirrhosis and 20% (n=10) were on treatment. 98% (n=49) were undergoing surveillance with 90% undergoing 12-monthly US. We analysed each patient's PAGE-B score from their most recent clinic data and 6 (12%) were stratified as high risk, 24 (48%) as medium risk and 20 (40%) as low risk. Of those coinfected with HIV, 15% (n=3) patients had cirrhosis and all patients were on anti-viral therapy. 7 patients (35%) were undergoing HCC surveillance. PAGE-B scores stratified 5 patients (25%) as high risk, 10 (50%) as medium risk and 5 (25%) as low risk. [Table 1 and 2]

**Figure:**
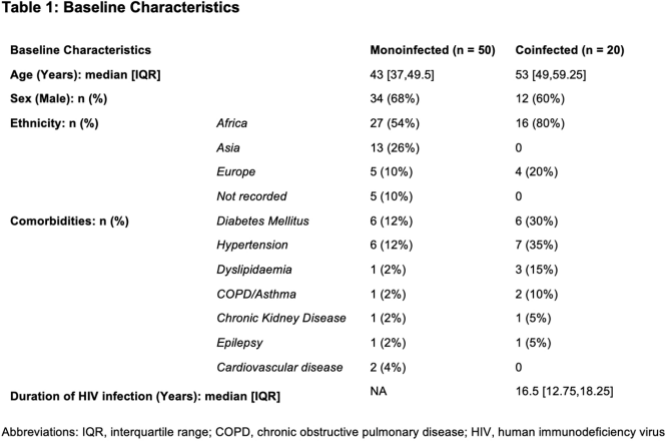

**Figure:**
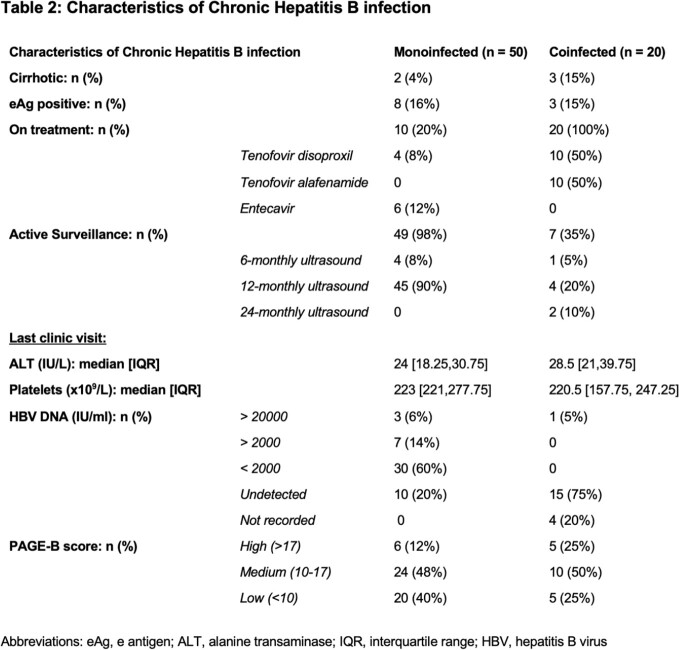

**Conclusion:**

The majority of patients with CHB monoinfection are receiving HCC surveillance with annual US. Using the PAGE-B score we identified 40% that are low risk and for which regular surveillance may be deferred. Those who were stratified as medium or high risk would benefit from more frequent 6-monthly US. This audit highlights the benefit of risk stratification in the allocation of resources. There was a lack of surveillance in our coinfected population despite 15 patients (75%) being stratified as medium or high risk. This may be due to clinicians focusing on HIV-associated issues during clinic visits and highlights the importance of addressing both infections when reviewing these patients.

**Disclosures:**

**All Authors**: No reported disclosures

